# Identification and Characterisation of a Novel Pathogenic Mutation in the Human Lipodystrophy Gene *AGPAT2*: C48R: A Novel Mutation in AGPAT2

**DOI:** 10.1007/8904_2012_181

**Published:** 2012-10-21

**Authors:** N. Ramanathan, M. Ahmed, E. Raffan, C. L. Stewart, S. O’Rahilly, R. K. Semple, H. Raef, J. J. Rochford

**Affiliations:** 01811 Institute of Metabolic Science, Addenbrooke’s Hospital, University of Cambridge Metabolic Research Laboratories, Hills Road, Cambridge, CB2 0QQ UK; 01812 Department of Medicine, King Faisal Specialist Hospital and Research Centre, 3354, Riyadh, 11211 Saudi Arabia; 01813 Institute of Medical Biology, Immunos, 8A Biomedical Grove, 138648 Singapore, Republic of Singapore

**Keywords:** Phosphatidic Acid, Single Amino Acid Substitution, Acanthosis Nigricans, Exome Sequencing Project, Congenital Generalise Lipodystrophy

## Abstract

Loss-of-function mutations in *AGPAT2*, encoding 1-acylglycerol-3-phosphate-O-acyltransferase 2 (AGPAT2), produce congenital generalised lipodystrophy (CGL). We screened the *AGPAT2* gene in two siblings who presented with pseudoacromegaly, diabetes and severe dyslipidaemia and identified a novel mutation in *AGPAT2* causing a single amino acid substitution, p.Cys48Arg. We subsequently investigated the molecular pathogenic mechanism linking both this mutation and the previously reported p.Leu228Pro mutation to clinical disease. Wild-type and mutant AGPAT2 were expressed in control and AGPAT2-deficient preadipocyte cell lines. mRNA and protein expression was determined, and the ability of each AGPAT2 species to rescue adipocyte differentiation in AGPAT2-deficient cells was assessed. Protein levels of both p.Cys48Arg and p.Leu228Pro AGPAT2 were significantly reduced compared with that of wild-type AGPAT2 despite equivalent mRNA levels. Stable expression of wild-type AGPAT2 partially rescued adipogenesis in AGPAT2 deficient preadipocytes, whereas stable expression of p.Cys48Arg or p.Leu228Pro AGPAT2 did not. In conclusion, unusually severe dyslipidaemia and pseudoacromegaloid overgrowth in patients with diabetes should alert physicians to the possibility of lipodystrophy. Both the previously unreported pathogenic p.Cys48Arg mutation in AGPAT2, and the known p.Leu228Pro mutation result in decreased AGPAT2 protein expression in developing adipocytes. It is most likely that the CGL seen in homozygous carriers of these mutations is largely accounted for by loss of protein expression.

## Introduction

Congenital generalised lipodystrophy (CGL) is a rare autosomal recessive disease characterised by nearly global loss of adipose tissue from birth. Affected individuals are commonly severely dyslipidaemic, insulin resistant, and eventually diabetic and suffer a commensurately high rate of microvascular and macrovascular complications, as well as a high rate of severe fatty liver disease and pancreatitis. Like many other patients with extreme forms of insulin resistance, they may also exhibit acromegaloid soft tissue overgrowth without any evidence of growth hormone or IGF1 excess. Although the diagnosis of CGL is usually made based on the observation of absent adipose tissue on clinical examination, the severity of the complications and collateral clinical and biochemical features may serve to alert clinicians to the underlying diagnosis.

Defects in four genes – *AGPAT2, BSCL2, CAV1*, and *PTRF* – have been implicated in CGL to date (OMIM: 608594, 269700, 612526, and 613327, respectively) (Rochford 2010). *AGPAT2*, the first of these to be reported, was implicated first by genetic mapping of the disease locus to chromosome 9q34, followed by identification of the disrupted gene in 2002 (Agarwal et al. 2002). Patients with *AGPAT2* mutations fail to develop metabolically active adipose tissue in most subcutaneous, intra-abdominal, intra-thoracic, and bone marrow fat depots, as ascertained by whole-body magnetic resonance imaging. However, they often have preserved mechanical adipose tissue depots in the palms, soles, scalp, retro-orbital, and peri-articular regions (Agarwal et al. 2003).

*AGPAT2* encodes a 278 amino acid acyltransferase, 1-acylglycerol-3-phosphate-O-acyltransferase 2 (AGPAT2) that converts lysophosphatidic acid (LPA) to phosphatidic acid (PA), a key step in the triacylglycerol (TG) biosynthesis pathway (Shindou et al. 2009). AGPAT2 is highly expressed in human adipose tissues, liver, pancreas, skeletal muscles, and small intestine (Shindou et al. 2009). Most reported *AGPAT2* mutations in CGL cause frame-shifts, insertions, deletions, or affect splicing, and are predicted to be functionally null alleles. Only a small number of pathogenic amino acid substitutions have been described to date (Hegele et al. 2007; Huang-Doran et al. 2010; Pelosini et al. 2011). In this report, we describe and characterise a novel missense mutation in the poorly characterised amino terminal domain of AGPAT2, p.Cys48Arg, identified in two female patients with CGL.

## Methods

### Mutational Analysis

Genomic DNA was extracted from peripheral blood leukocytes before PCR amplification of all coding exons of the *AGPAT2* and *BSCL2* genes plus 50 bp of flanking sequence at either end of the exons employing primers designed using ExonPrimer via the USC genome browser (http://genome.ucsc.edu/) and checked for common polymorphisms. The PCR products were purified using Agencourt® CleanSEQ® reagents (Agencourt Bioscience, Beverly, MA, USA) and sequenced in both directions using the BigDye® Terminator v3.1 Cycle Sequencing Kit (Applied Biosystems, PerkinElmer, Foster City, CA, USA) and an ABI 3730 DNA sequencer (Applied Biosystems). Sequence analysis was performed using Sequencher software (Gene Codes, Ann Arbor, MI).

### Biochemical Assays

Insulin, leptin, and adiponectin were all assayed on a 1235 AutoDELFIA (PerkinElmer Lifesciences, Boston, MA) automatic immunoassay system using two-step time-resolved fluorometric assays as previously described (Semple et al. 2006). SHBG was determined using an IMMULITE 1,000 solid phase chemiluminescent enzyme immunometric assay (Siemens Medical Solutions Diagnostics). Testosterone was measured by a solid phase extraction RIA (Siemens Healthcare Diagnostics, Surrey, UK; formally DPC Coat-A-Count) using protocols provided by the manufacturer. Triglyceride concentrations were assayed in singleton on the Dimension RXL system (Dade Behring, Milton Keynes, UK). Plasma (total) IGF-I concentrations were measured in ethanolic extracts using aDSLELISA (DSL-UK Ltd., Upper Heyford, Oxon, UK) according to the manufacturer’s instructions.

### Cell Culture

C3H10T1/2 murine mesenchymal stem cells and 3T3-L1 preadipocytes were grown in Dulbecco’s modified Eagle’s medium (DMEM) containing 10 % newborn calf serum (NCS) as previously described (Payne et al. 2008). shRNA sequences targeting AGPAT2 were designed and cloned into the RNAi-Ready pSIREN-RetroQ vector (Clontech) and shRNA targeting luciferase used as a control. Wild-type human AGPAT2 was cloned into pcDNA3.1myc-HisA vector (Invitrogen) and p.Cys48Arg and p.Leu228Pro mutant forms created by site directed mutagenesis. Myc-tagged AGPAT2 constructs were subcloned into the pBabe retroviral vector. Retroviruses carrying shRNA or cDNA constructs were used to infect preadipocyte cell lines as previously described (Payne et al. 2008). Oil Red O staining of lipid accumulation was as described in (Payne et al. 2008).

### Western Blot Analysis

Cells were scraped in lysis buffer (50 mM Tris pH6.8, 150 mM NaCl, 1mM EDTA, Complete Protease Inhibitor Cocktail (Roche), PhosSTOP Phosphatase Inhibitor Cocktail (Roche), and 50 mM n-β-d-glucopyranoside (Calbiochem)), sonicated, centrifuged at 14,000*g* for 10 min and protein concentration determined using a (BCA) kit (Bio-Rad). Samples containing equal amounts of protein were resolved on a NuPAGE® Novex® 4–12 % Bis-Tris precast gels (Invitrogen) and transferred using iBlot® (Invitrogen). Membranes were blocked in 5 % milk then probed with anti-myc (mouse monoclonal 4A6) (Milipore) or anti-calnexin antibodies. Secondary antibodies were from Thermo Scientific and signals were detected using ECL (GE Healthcare).

### Real Time PCR

Cells were collected and RNA isolated using an RNAeasy kit (Qiagen). Purified RNA was reverse transcribed using random hexamers and M-MLV reverse transcriptase (Promega). Gene expression was assayed by qPCR using either TaqMan® primers and probes (ABI Biosystems) or SYBR green and normalised to cyclophilin A.

### Case Histories

This study was conducted in accordance with the Declaration of Helsinki and approved by the UK national research ethics committee. Written informed consent was obtained from both participants.

**Fig. 1:**
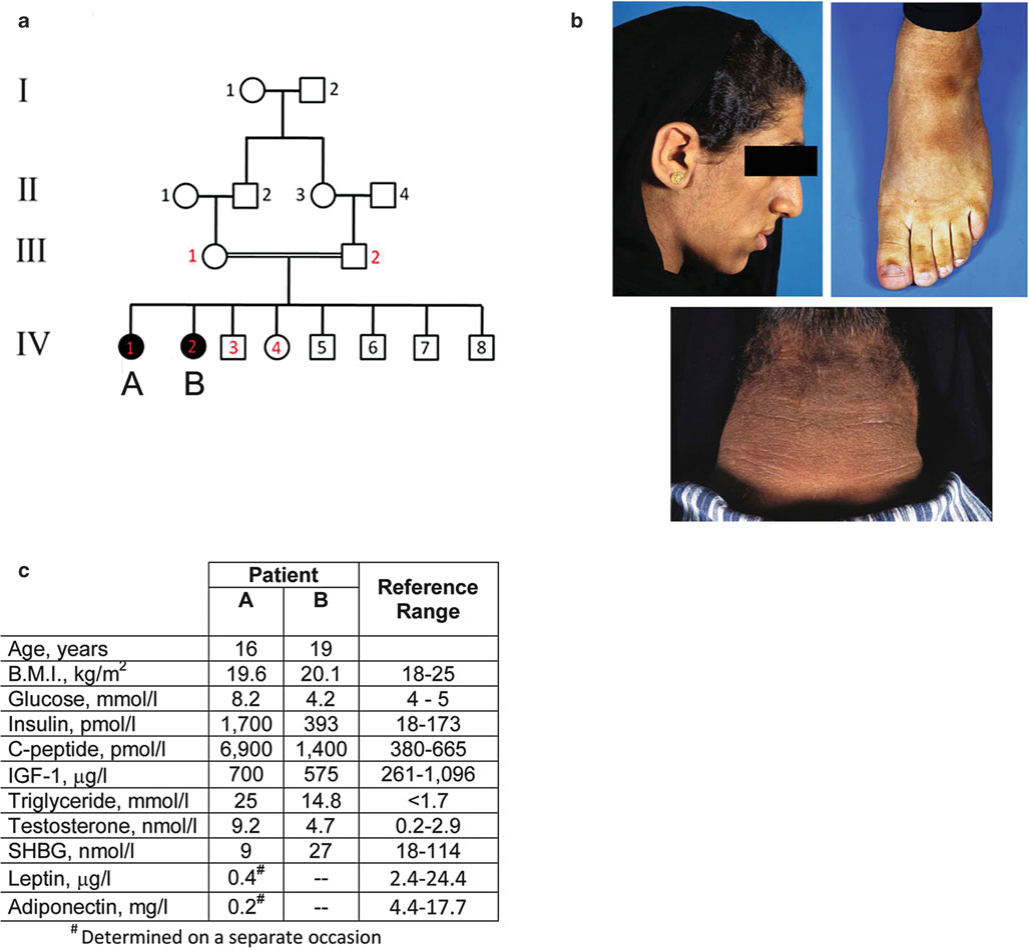
(**a**) Family pedigree of the lipodystrophic patients in this study. Patients A and B are homozygous for the AGPAT2 p.Cys48Arg mutation, while family members in red are heterozygotes. (**b**) Appearance of patient A, showing acromegaloid overgrowth (*upper left image*) and acanthosis nigricans of the foot (*upper right image*) and nuchal acanthosis nigricans (*lower image*). (**c**) Biochemical profile of the patients studied

*Patient A* (14 years old at presentation) and her sister *patient B* (17 years old), both from Saudi Arabia, presented complaining of a male habitus and oligomenorrhoea. Their parents were first cousins (Fig. 1a). Both sisters were lean with marked hirsutism, androgenetic alopecia, deep voices, and muscle hypertrophy. They also had acanthosis nigricans with acrochordons (Fig. 1b), a markedly acromegaloid facial appearance (Fig. 1b), relatively large hands and feet, and although a paucity of subcutaneous fat was also noted, it was these features of overgrowth that were the focus of the initial diagnostic evaluation. Both patients had severe hyperinsulinemia, hypertriglyceridemia, and hyperandrogenemia with low sex hormone binding globulin (Fig. 1c). Serum IGF-1 and growth hormone response to 75 g oral glucose, and 17 hydroxyprogesterone response to 250 mcg synthetic ACTH were normal. At 15 years old patient A was found to be diabetic with an HbAIC of 16.4 %, and metformin, glyburide, gemfibrozil, and simvastatin were commenced. Subsequently insulin was introduced and titrated to 166 units/day, along with a maximal dose of pioglitazone. Despite these measures proliferative retinopathy, nephropathy, neuropathy, and severe hepatic steatosis developed. At that stage serum adiponectin and leptin were nearly undetectable, in keeping with near complete absence of adipose tissue. Patient B developed diabetes at 18 years old and is currently taking metformin and pioglitazone with no complications to date.

## Results

**Fig. 2:**
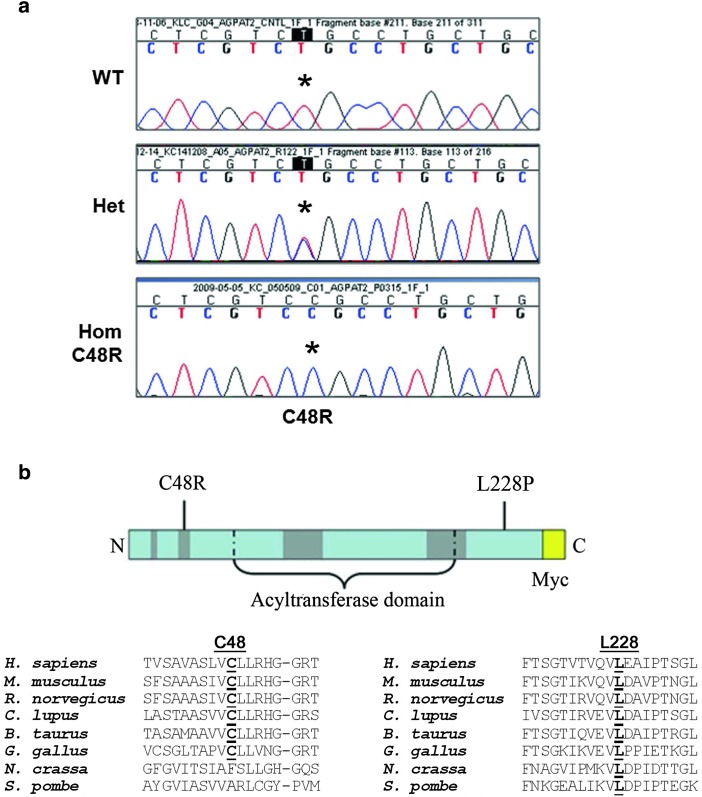
(**a**) Representative chromatograms from sequencing genomic DNA of wild-type individuals (*upper panel*) or heterozygote (*middle panel*) or homozygote (lower panel) carriers of the AGPAT2 p.Cys48Arg mutation. (**b**) Schematic diagram showing the location of the acyltransferase domain and the p.Cys48Arg and p.Leu228Pro mutations within AGPAT2. Transmembrane domains are shown in *grey*. Phylogenic conservation of Cys48 and Leu228 are shown below

No rare sequence variants were identified in the *BSCL2* gene; however, both affected probands were found to be homozygous for the *AGPAT2* c.142T>C transversion, producing the p.Cys48Arg missense change in the AGPAT2 protein (representative chromatogram Fig. 2a lower panel). Both parents, and two clinically unaffected siblings available for testing were heterozygous for the mutation (representative chromatogram Fig. 2a middle panel), which was absent from more than 900 control patients from a panel of different ethnicities. In addition, the variant was not found in the NHLBI ESP Exome Variant Server, including around 4,000 people drawn from various cohort studies (NHLBI Exome Sequencing Project Exome Variant Browser (ESP), Seattle, WA, URL: http://evs.gs.washington.edu/EVS/) accessed October 2011). Cysteine 48 is highly phylogenetically conserved (Fig. 2b) and falls within the second transmembrane domain of AGPAT2.

**Fig. 3:**
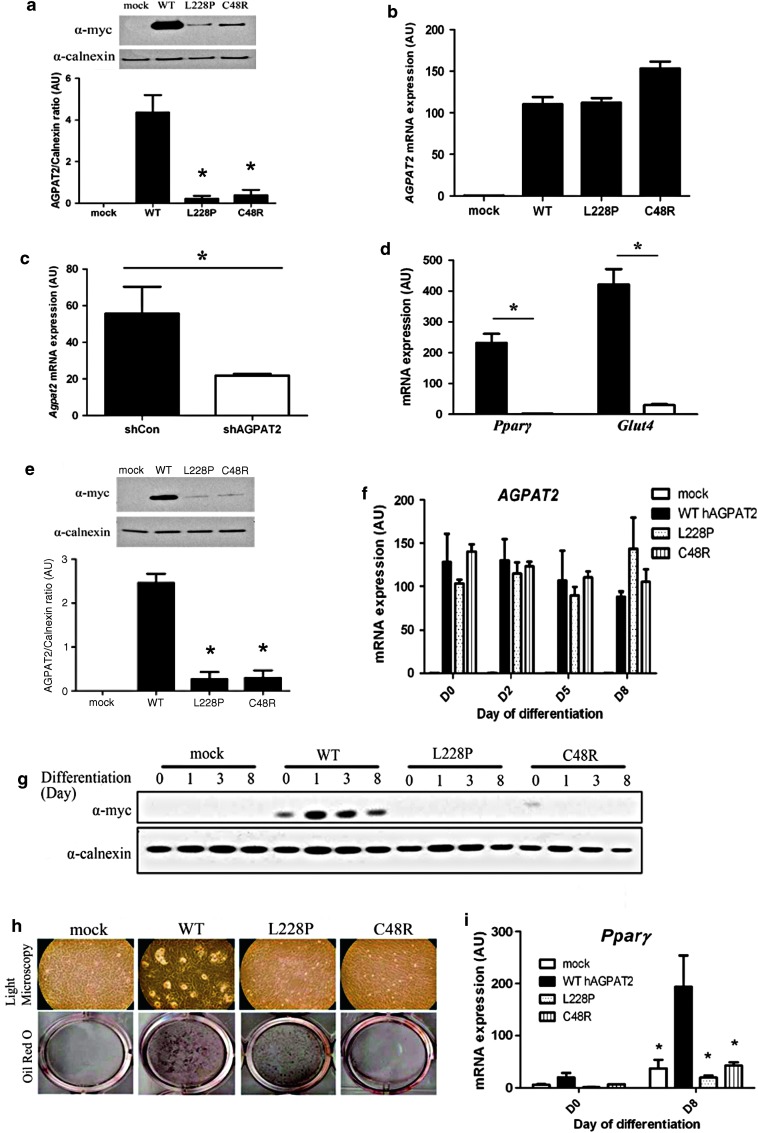
(**a**) Protein levels of myc-tagged AGPAT2 determined by immunoblotting in 3T3-L1 preadipocytes stably expressing wild-type (WT) Leu228Pro (L228P) or Cys48Arg (C48R) AGPAT2 compared to mock infected cells. Data shown are mean +/− SEM of three independent experiments normalised to calnexin expression in the same samples as a loading control, * indicates a difference of *p* < 0.05 compared with expression of the wild-type AGPAT2. A representative blot is shown above. (**b**) AGPAT2 mRNA levels in the same cells as (A) **(C)** AGPAT2 mRNA levels in murine C3H10 T1/2 cells with (shAGPAT2) or without (Con) stable expression of shRNA targeting murine AGPAT2. (**d**) Pparg2 and Glut4 mRNA levels after 8 days of adipogenic differentiation in C3H10T1/2 cells stably expressing either control shRNA (*black bars*) or murine AGPAT2 shRNA (*white bars*) (**e**) Protein levels of AGPAT2 after stable expression of human wild-type or mutant AGPAT2 in C3H10T1/2 cells with stable murine Agpat2 knockdown determined by immunoblotting. Data shown are mean +/− SEM of 3 independent experiments normalised to calnexin expression in the same samples as a loading control, * indicates a difference of *p* < 0.05 compared with expression of the wild-type AGPAT2. A representative blot is shown above. (**f**) human AGPAT2 mRNA levels in the cells in (E) following induction of adipogenesis for the times shown. (**g**) Protein levels of myc-tagged wild-type and mutant AGPAT2 in the same cells as in (**f**) during adipogenic differentiation, assessed by immunoblotting, with calnexin as a loading control. (**h**) Triglyceride accumulation assessed by light microscopy (*upper panels*) and Oil Red O staining (*lower panels*) in C3H10T1/2 cells with stable Agpat2 knockdown and human wild-type or mutant AGPAT2 re-expression after 8 days of adipogenic differentiation. (**i**) Real time PCR analysis of Pparγ mRNA expression in C3H10T1/2 cells with stable Agpat2 knockdown and human wild-type or mutant AGPAT2 re-expression at day 0 and after 8 days of adipogenic differentiation. All real time PCR data are shown +/−SEM, *n* = 3, * indicates difference of *p* < 0.05, versus expression in cells re-expressing wild-type AGPAT2 at the same time point determined using two-tailed paired Student’s *T* test. All western blots and cell images are representative of at least three independent experiments

To assess the effect of the AGPAT2 p.Cys48Arg mutation we generated 3T3-L1 murine preadipocytes stably expressing wild-type human AGPAT2, AGPAT2 Cys48Arg, or the previously described AGPAT2 Leu228Pro. The latter was selected as Leu228 is the most phylogenetically conserved of the residues reported to be substituted in CGL, being invariant in all species examined (Fig. 2b). This suggests that it may serve a particularly critical function in the mature AGPAT2 enzyme. Consistent with this AGPAT2 Leu228Pro has the lowest activity of any pathogenic single amino acid substitutions in AGPAT2 examined in a previous study (Haque et al. 2005). Western blotting revealed dramatically lower expression of the Leu228Pro and Cys48Arg mutant proteins compared to wild-type AGPAT2 in confluent preadipocytes (Fig. 3a). To investigate this further human AGPAT2 mRNA was determined using a quantitative real time PCR assay that did not detect murine AGPAT2 mRNA. As expected, no human AGPAT2 mRNA expression was detected in control cells whilst equal levels were present in cells expressing AGPAT2 wild-type, Leu228Pro, or Cys48Arg (Fig. 3b). The low expression of mutant AGPAT2 protein with normal mRNA levels suggests that both Leu228Pro and Cys48Arg AGPAT2 mutations most likely affect AGPAT2 folding and/or stability.

In order to model the in vivo situation in lipodystrophic patients more faithfully the effect of expressing wild-type or mutant AGPAT2 in murine preadipocytes with stable Agpat2 knockdown was examined. Murine Agpat2 was selectively knocked down in the C3H10T1/2 mesenchymal stem cell line using retroviruses expressing either control shRNA or mouse-specific shRNA against Agpat2. As shown in Fig. 3c, the low levels of Agpat2 present in undifferentiated cells were significantly reduced by expression of shRNA targeting murine Agpat2. When Agpat2 knockdown cells were induced to differentiate for 8 days in culture they showed significantly impaired expression of the key adipogenic transcription factor PPARγ and the insulin-sensitive glucose transporter Glut4, both well-characterised markers of adipogenesis (Fig. 3d). Human wild-type Leu228Pro or Cys48Arg AGPAT2 were then overexpressed stably using a second retroviral vector. As in 3T3-L1 cells, expression of Leu228Pro and Cys48Arg AGPAT2 proteins was significantly reduced compared to wild-type (Fig. 3e). This was despite equivalent expression of wild-type or mutant AGPAT2 mRNAs both as confluent preadipocytes and at various time points following induction of differentiation in these cells (Fig. 3f). Moreover the Leu228Pro and Cys48Arg AGPAT2 proteins remained undetectable at various time points tested following adipogenic induction (Fig. 3g). Consistent with this, expression of wild-type AGPAT2 partially rescued adipogenesis in AGPAT2 knockdown cells whereas neither Leu228Pro nor Cys48Arg mutant AGPAT2 constructs were able to do so when assessed by either light microscopy or oil red O staining of lipid accumulation (Fig. 3h) or by assessment of the induction of Pparγ mRNA expression (Fig. 3i).

## Discussion

CGL is usually recognised in infancy due to severe lack of adipose tissue. However, these cases are a reminder that in older patients collateral features of lack of adipose tissue, including severe dyslipidaemia and fatty liver disease, and the hyperandrogenic and pseudoacromegalic features of severe insulin resistance, may instead dominate the clinical presentation. Thus, each of these should serve as ‘red flags’ alerting physicians to possible underlying lipodystrophy.

We investigated the effect of a novel pathogenic mutation in AGPAT2, p.Cys48Arg, and a previously described mutation, p.Leu228Pro, on the function of AGPAT2. Both mutations led to dramatically reduced protein expression suggesting that this may be the dominant reason for failure of adipose tissue formation in these patients. As the mutant forms of AGPAT2 were very poorly expressed it would not be trivial to assess their enzymatic activity accurately. As such we cannot formally exclude the possibility that, should these proteins be expressed in the affected individuals, they might also display reduced enzymatic activity. Whilst it may seem surprising that a single amino acid substitution so dramatically affects protein expression this has been observed with pathogenic mutations in other proteins such as steroid 5β-reductase (Mindnich et al. 2011). As the p.Cys48Arg mutation occurs in the putative second transmembrane domain of AGPAT2 it is possible that the mutation causes misfolding of this domain and altered membrane insertion, leading to degradation. However, a fuller understanding of the structure of AGAPT2 and further studies including modelling of this mutation would be required to determine this. A previous study of several pathogenic mutations in AGPAT2 including p.Leu228Pro (Haque et al. 2005) reported that Leu228Pro AGPAT2 exhibited significantly lower AGPAT activity than wild-type AGPAT2. However, critically, the expression of wild-type AGPAT2 was not directly assessed in parallel. Given our findings that both p.Leu228Pro and p.Cys48Arg are expressed at significantly reduced levels, it will be interesting to determine whether this may also be so with other pathogenic mutants of AGPAT2 found in CGL patients.

The precise mechanism whereby AGPAT2 affects the complex process of adipogenesis remains unclear. However, it is evident that AGPAT2 deficiency does not merely result in the selective loss of triglyceride synthesis. Knockdown of AGPAT2 expression in cultured preadipocytes results in marked inhibition of adipogenic gene expression (Gale et al. 2006). The recent demonstration that, as in humans, Agpat2 deficiency in mice produces generalised lipodystrophy and severe insulin resistance provides an in vivo model for further investigations (Cortes et al. 2009). AGPAT2 loss may alter the generation of lipid species that can directly or indirectly play a role in modulating gene transcription. PA, the product of AGPAT2 activity, is a key intermediate in the production of several phospholipids which may influence both biogenesis and/or intracellular signalling. Interestingly, AGPAT2 inhibition in vascular smooth muscle cells suppresses the activation of PI3-kinase/AKT and MAPK pathways, both known to have roles in adipocyte differentiation (Coon et al. 2003). Evidently, further studies will be required to elucidate which, if any of these pathways, may be influenced by AGPAT2 during adipocyte development.

This study is the first to rescue adipogenesis in cultured cells lacking AGPAT2 by transfection with the wild-type enzyme and to demonstrate that expression of pathogenic mutants of AGPAT2 cannot do this. In the case of the mutants examined here this resulted from dramatically reduced expression of the mutants. However, it demonstrates that this system may be valuable for studying the underlying pathogenic mechanism of other mutants where protein expression is not affected.

In conclusion, we report the novel Cys48Arg pathogenic mutation in AGPAT2 and show that this and the previously described Leu228Pro mutation lead to dramatically reduced protein expression. Whilst we cannot exclude the possibility that Cys48Arg and Leu228Pro forms of AGPAT2 may also exhibit reduced enzymatic activity, we suggest that reduced expression of these mutated proteins may significantly contribute to the CGL phenotype seen in these patients.

## Summary

Loss-of-function mutations in *AGPAT2*, encoding 1-acylglycerol-3-phosphate-O-acyltransferase 2 (AGPAT2), produce congenital generalised lipodystrophy (CGL). We screened the *AGPAT2* gene in two siblings who presented with pseudoacromegaly, diabetes, and severe dyslipidaemia and identified a novel mutation in *AGPAT2* causing a single amino acid substitution, p.Cys48Arg. We subsequently investigated the molecular pathogenic mechanism linking both this mutation and the previously reported p.Leu228Pro mutation to clinical disease. Wild-type and mutant AGPAT2 were expressed in control and AGPAT2-deficient preadipocyte cell lines. mRNA and protein expression were determined, and the ability of each AGPAT2 species to rescue adipocyte differentiation in AGPAT2-deficient cells was assessed. Protein levels of both p.Cys48Arg and p.Leu228Pro AGPAT2 were significantly reduced compared with that of wild-type AGPAT2 despite equivalent mRNA levels. Stable expression of wild-type AGPAT2 partially rescued adipogenesis in AGPAT2 deficient preadipocytes whereas stable expression of p.Cys48Arg or p.Leu228Pro AGPAT2 did not. In conclusion, unusually severe dyslipidaemia and pseudoacromegaloid overgrowth in patients with diabetes should alert physicians to the possibility of lipodystrophy. Both the previously unreported pathogenic p.Cys48Arg mutation in AGPAT2, and the known p.Leu228Pro mutation result in decreased AGPAT2 protein expression in developing adipocytes. It is most likely that the CGL seen in homozygous carriers of these mutations is largely accounted for by loss of protein expression.

## Synopsis

We have identified a novel mutation in the gene *AGPAT2* causing generalised lipodystrophy and characterised this in cultured models of adipocyte development which suggest that the mutation results in a failure to express the protein and a consequent lack of adipogenesis.

## Conflict of Interest Statement

The authors declare that they have no conflict of interest relevant to the study reported in this manuscript.
